# Structural, Compositional, and Mechanical Characterization of W_x_Cr_y_Fe_1−x−y_ Layers Relevant to Nuclear Fusion, Obtained with TVA Technology

**DOI:** 10.3390/ma12244072

**Published:** 2019-12-06

**Authors:** Mihail Lungu, Ioana Porosnicu, Paul Dinca, Alin Velea, Flaviu Baiasu, Bogdan Butoi, Oana Gloria Pompilian, Cornel Staicu, Parau Anca Constantina, Corneliu Porosnicu, Cristian Lungu, Ion Tiseanu

**Affiliations:** 1 Low Temperature Plasma and Plasma Physics and Nuclear Fusion Departments, National Institute for Lasers, Plasma and Radiation Physics, 077125 Bucharest, Romania; mihail.lungu@inflpr.ro (M.L.); ioana.porosnicu@inflpr.ro (I.P.); flaviu.baiasu@inflpr.ro (F.B.); bogdan.butoi@inflpr.ro (B.B.); oana.pompilian@inflpr.ro (O.G.P.); cornel.staicu@inflpr.ro (C.S.); corneliu.porosnicu@inflpr.ro (C.P.); cristian.lungu@inflpr.ro (C.L.); ion.tiseanu@inflpr.ro (I.T.); 2 Faculty of Physics, University of Bucharest, 405 Atomistilor Street, 077125 Magurele, Romania; 3 Nanoscale Condensed Matter Department, National Institute for Material Physics, 077125 Bucharest, Romania; alin.velea@infim.ro; 4 Advanced Surface Processing and Analysis by Vacuum Technologies Department, National Institute for Optoelectronics, 077125 Bucharest, Romania; anca.parau@inoe.ro

**Keywords:** TVA method, W_x_Cr_y_Fe_1−x−y_ layers, EUROFER, micro-XRF

## Abstract

Reduced activation ferritic and martensitic steel like EUROFER (9Cr-1W) are considered as potential structural materials for the first wall of the future next-generation DEMOnstration Power Station (DEMO) fusion reactor and as a reference material for the International Thermonuclear Experimental Reactor (ITER) test blanket module. The primary motivation of this work is to study the re-deposition of the main constituent materials of EUROFER, namely tungsten (W), iron (Fe), and chromium (Cr), in a DEMO type reactor by producing and analyzing complex W_x_Cr_y_Fe_1−x−y_ layers. The composite layers were produced in laboratory using the thermionic vacuum arc (TVA) method, and the morphology, crystalline structure, elemental composition, and mechanical properties were studied using scanning electron microscopy (SEM), X-ray diffraction (XRD), micro-X-ray fluorescence (micro-XRF), and glow discharge optical emission spectrometry (GDOES), as well as nanoindentation and tribology measurements. The results show that the layer morphology is textured and is highly dependent on sample positioning during the deposition process. The formation of polycrystalline W_x_Cr_y_Fe_1−x−y_ was observed for all samples with the exception of the sample positioned closer to Fe anode during deposition. The crystalline grain size dimension varied between 10 and 20 nm. The composition and thickness of the layers were strongly influenced by the in-situ coating position, and the elemental depth profiles show a non-uniform distribution of Fe and Cr in the layers. The highest hardness was measured for the sample positioned near the Cr anode, 6.84 GPa, and the lowest was 4.84 GPa, measured for the sample positioned near the W anode. The tribology measurements showed an abrasive sliding wear behavior for most of the samples with a reduction of the friction coefficient with the increase of the normal load.

## 1. Introduction

In the last 50 years, the fast development of civilization combined with the need for reducing the CO_2_ emissions worldwide determined an increasing demand for a cleaner energy, both in the household and in the industrial sector. Researchers worldwide make considerable efforts to further develop new types of energy sources in order to overcome the need for a cleaner energy and to reduce today’s growing dependence on fossil fuels [1]. Until now, the nuclear fusion energy was established to have the potential to provide huge benefits to society, thus creating a Sun-like power generation source on Earth. The knowledge in the nuclear physics area expanded with the development of “tokamak-type” experimental fusion reactors like the Joint European Torus and Tokamak Fusion Test Reactor (TFTR) [2]. One of the several challenges regarding the production of fusion energy on Earth is represented by the determination of materials to be integrated in the blanket modules and as plasma facing components (PFC). Particularly, the future design of the International Thermonuclear Experimental Reactor relies on the selection of the proper materials as first-wall materials [3]. Therefore, it is crucial to improve the functionality and design of the PFC to ensure the future of fusion reactors like DEMO (DEMOnstration Power Station) and their economic reliability and benefits [4]. The research was conducted on different types of materials such as steel, which represents the most promising solution in order to determine cheap and reliable materials to be integrated as PFC. One of the most promising materials is represented by the reduced activation ferritic martensitic steel (RAFM) [5,6,7,8,9]. The RAFM steel such as EUROFER (9Cr-1W) [5,9] is a candidate material for future DEMO reactors [10] and ITER’s test blanket module (TBM). EUROFER 97 has an elemental composition which comprises mostly of 89.3 at% Fe, 9.2 at% Cr, 1.2 at% W, and 0.3 at% other elements (C, V, Mn, and Ta). It is a reference material fabricated in Europe under the European Fusion Development Agreement (EFDA)-Structural Materials to be used for DEMO type fusion reactors. It exhibits important properties like reduced neutron activation, good permeability, low hydrogen isotope retention, and good thermo-conductive properties up to 943 K [11,12]. Due to extreme heat loads expected in the divertor area of the tokamak, up to 20 MW/m^2^, the use of RAFM steel will be limited to the first wall which will be subjected to lower heat (up to 1 MW/m^2^) and neutron and ion fluxes [13]. One of the key issues in today’s experimental fusion reactions is represented by material migration, transport, and co-deposition due to plasma–wall interaction conditions. For example, in JET-ILW (equipped like ITER with a first Be wall and a W divertor) considerably thick Be co-deposits were observed on the inner divertor wall [14,15]. The same behavior will most likely occur in DEMO type reactors. Poor adhesion of the co-deposited layers on the W substrates can severely influence the fusion plasma and fusion fuel (^3^H) inventory due to flaking of co-deposited layers under high heat loads and particle fluxes resulting in dust formation. 

Therefore, the main purpose in the current experimental study was the investigation of the structural, compositional, and mechanical properties of W_x_Cr_y_Fe_1−x−y_ composite layers similar to EUROFER97, co-deposited on W substrates. This study is also intended to validate Thermionic Vacuum Arc (TVA) as a suitable deposition method for fusion related materials, and the protocol for the powerful, fast, and reliable tool of the XRF method assisted with the XRF-FP (X-ray fluorescence fundamental parameters) software (version 6.0, CrossRoads Scientific, Middletown, MA, USA) was successfully implemented. 

## 2. Materials and Methods

### 2.1. Samples Co-Deposition Method

The W_x_Cr_y_Fe_1−x−y_ layers that present a high interest in the nuclear fusion domain were co-deposited by means of the TVA method. This technique was also used to obtain mixed Be-W and Be-C layers characterized by high purity, good substrate adhesion, and controlled atomic composition confirmed by several studies [16,17,18,19,20,21]. The method implies a co-deposition process by means of three anode-cathode systems, where the cathode consists of a circular heated W filament integrated as an electron gun. The thermo-emitted electrons are focused on the anode with the help of a grounded Wehnelt cylinder that has the role of an electrostatic lens. The anode consists of the materials that will be deposited, and it is placed in crucibles made from a highly thermally resistant alloy, such as titanium diboride (TiB_2_). For highly refractory materials, as is the case for W, the anode consists of a W rod.

**Figure 1:**
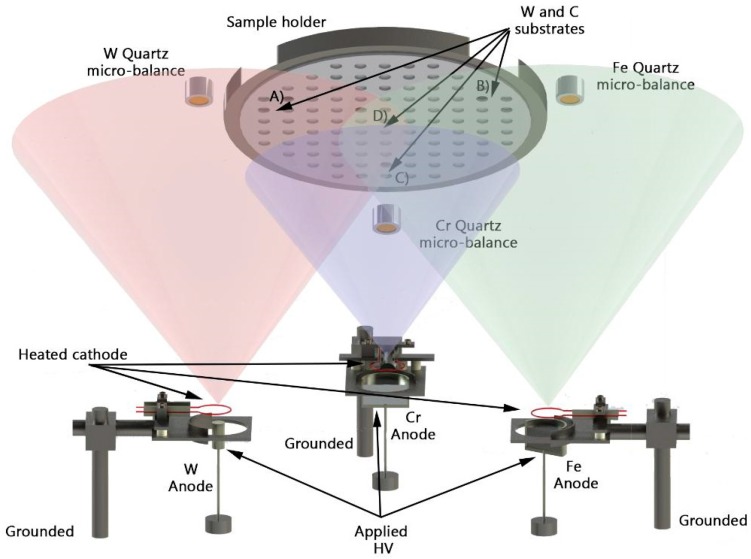
Thermionic vacuum arc (TVA) experimental setup that integrates three anode-cathode systems for the W-Fe-Cr sample co-deposition campaign. The position of the samples investigated in this study is marked on the sample holder.

By applying a high voltage in the order of several kV, the anode material is heated by the accelerated electrons. This process leads to the evaporation of the anode material and ultimately ignites the plasma in the metallic vapors of the deposited material. It is important to add that the processes mentioned above takes place in high vacuum conditions (10^−4^ Pa). In this experimental study, three anode-cathode systems were used, and they were operated individually, placed in the vacuum chamber, and equally spaced from each other in a triangular geometry. The distance between the systems was 30 cm in order to prevent for one anode-cathode system to influence the discharge parameters of another system. The involved experimental setup for the W_x_Cr_y_Fe_1−x−y_ co-deposition is illustrated in Figure 1.

The anode used for W deposition consists of a 10 mm diameter rod with a high purity reaching 99.95%. The W anode was placed at 24 cm from the sample substrate, and the distance between anode and the quartz micro-balance was also 24 cm. Cr deposition material consisted of pieces with dimensions of 0.8–6 mm and a 99.95% purity placed at a distance of 31.5 cm from the sample substrate and also from the monitor balance. The Fe anode consisted of 6.35 mm diameter and 6.35 mm length iron pellets with 99.95% purity placed at the same distance as mentioned for the Cr anode. Both Cr and Fe powders were placed in TiB_2_ crucibles. All the precursor materials were purchased from Kurt J. Lesker Company (Hastings, East Sussex, England). The main relevant parameters of the discharge, such as the anodic voltage (U_a_), the discharge current (I_a_), and the filament current (I_f_), are provided in Table 1. The total deposited thickness was calculated for the central area of the sample holder based on measurements from all film thickness monitors (FTM7 manufactured by Edwards High Vacuum, Crawley, Sussex, England) with micro-quartz balance.

The substrates consisted of 12 mm × 15 mm highly polished C (average roughness is lower than 120 nm) and W (average roughness is lower than 500 nm) materials. A total number of eight samples was obtained applying the above-mentioned coating process: four samples on C substrates and four samples that involved the W substrate. A cleaning procedure was performed in order to obtain the best adherence of the coated layers on the substrate. These were cleaned with a mixture of acetone and isopropyl alcohol in an ultrasonic bath to remove the surface impurities. After the cleaning process, the substrates were mounted in a circular holder that is places in the middle of the chamber at a distance of 25 cm from the plasma evaporation sources. Two samples were integrated in the middle of the sample holder in order to obtain the elemental concentration similar to EUROFER 97, namely W 2%, Cr 9%, and Fe 89%. Since the re-deposition of materials in a nuclear fusion reactor is not a stoichiometric process, due to different erosion yields of materials, each type of substrate was placed in the holder at normal incidence above each plasma source. In this manner, different elemental composition was obtained in the same experimental coating campaign. During the coating process, different evaporation rates were used for each element in order to achieve the desired layer composition. In this work, the evaporation rates and layer thickness were motorized in-situ with an FTM7 micro-quartz balance monitor (QMB, Edwards High Vacuum, Crawley, Sussex, England) (Table 1). 

The total co-deposited thickness layer, by summing the contributions of W, Fe, and Cr, was 2877 nm. Sample identification relies on their position in holder, during the coating process, in relation to the evaporation sources (Table 2).

### 2.2. Methods of Investigation

Elemental composition analysis was conducted by means of the XRF method enhanced with the fundamental parameter theoretical approach. Thus, calibrated measurements of layer stoichiometry on co-deposited W-Fe-Cr composite layers were achieved. The micrometer resolution of the XRF method is achieved by integrating an optical collimating system. Therefore, the method will be further named micro-XRF, with an investigation diameter spot of tens of micrometers (~30 µm). The micro-XRF method has multiple advantages based on its simplicity, small range focal spot size, fast operation, lack of moving parts, and high source yield. This proprietary in-house-built method implies the use of an X-ray excitation source working at low energies (<50 keV) and a Si-pin energy selective detector.

SEM images were acquired in order to investigate the surface morphology of the coated layer. With this method, the wear mechanisms were also studied through the analysis of the erosion tracks produced during tribological measurements. The SEM images were taken with a FEI Co. model Inspect S. instrument (Hillsboro, OR, USA) using a working pressure of 1.5 × 10^−2^ Pa and at distance of 11.9 mm, with a 20 kV acceleration voltage and a magnification of 20,000× and 5000× for sample morphology and erosion patterns analysis, respectively.

Structural investigations were performed on W_x_Cr_y_Fe_1−x−y_ layers deposited on C substrates by means of X-ray diffraction (XRD). Measurements were performed with a Bruker D8 Advance diffractometer (Coventry, West Midlands, England) provided with a Cu-K_α_ X-ray source, with a specific wavelength of 0.154178 nm and Lynx Eye detector. Measurement was performed in 2θ range between 40° and 120° with a step size of 0.05° and 200 s integration time per step.

Since the layer thickness is in the order of few micrometers, an accurate measurement of the hardness and elastic modulus using the classical micro-indentation method could provide erroneous results due to the substrate contribution. Therefore, in order to obtain accurate and correct results the nanoindentation method was applied. The nanomechanical properties of W, Fe, Cr coatings were investigated with a Berkovich indenter (Fischer-Cripps Lab. Pty. Limited, Model B, New South Wales, Australia), consisting of a pyramid with three faces angled at 65.311°. The load–unload measurements were performed at 4 mN to determine the hardness and elastic modulus of the coatings. The nanoindentations were performed on random areas of the samples. The selection of suitable spots to perform nanoindentation proved to be especially difficult due to the apparent surface roughness. After a series of 10 measurements was performed, three matching curves were selected, and the obtained results were averaged. Based on these results and by means of the Olivier and Pharr method, the values of the hardness and elastic modulus were determined.

Additionally, information regarding the sliding wear behavior of the deposited layers was provided using a ball-on-disk CMS tribometer (CSM instruments, Needham, MA, USA). The measurements were performed by sliding the sample surface against a steel ball with a linear speed of 2 cm at a normal load of 1, 3, and 5 N. The travel distance of the stainless-steel ball on each sample was set to 50 m.

The glow discharge optical emission spectrometry (GDOES) method was applied in order to provide elemental depth profiles through the deposited layer; Spectruma GDA 750 GDOES equipment (Spectruma Analytik GMBH, Hof, Germany) was used in this respect. The GDOES can be used to measure the chemical composition of materials in “bulk” or thin film profiling mode, thus quantifying the relation between elemental concentration and sample depth. The system is provided with 29 channels intended to measure the chemical concentration for 27 chemical elements. Therefore, both metallic and dielectric samples can be measured. The GDOES proved to be suitable for the analysis of WFeCr coatings due to its capability to measure coatings with thicknesses over 100 µm with a 1 nm range resolution in depth and with a detection limit of up to 0.1 ppm. Due to chamber contamination reasons, the GDOES method was applied only for investigating layers deposited on W substrate.

## 3. Results and Discussions

### 3.1. Structural and Morphological Analysis

**Figure 2:**
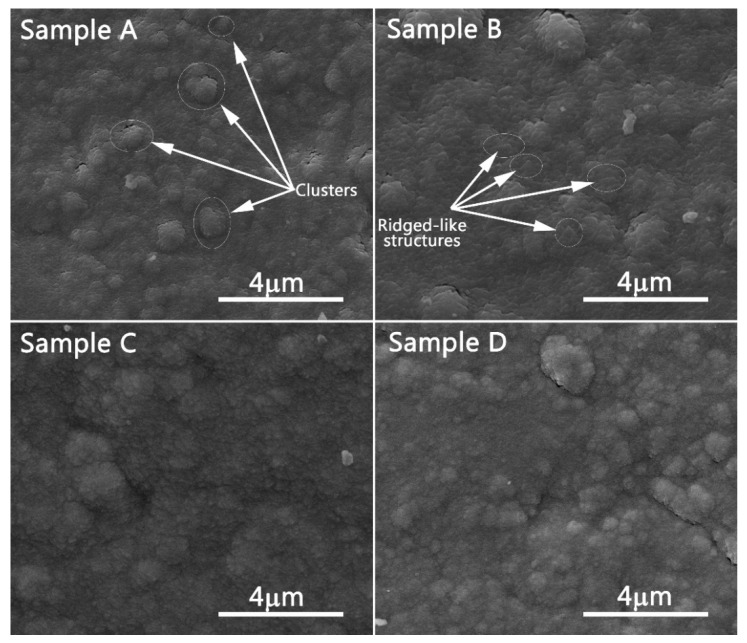
SEM images of W-Fe-Cr samples.

The SEM images of W-Fe-Cr samples deposited in fixed geometry are presented in Figure 2. They correspond to the samples placed during the deposition above each anode and those placed in the center of the geometry, respectively. Only the samples coated on W substrate were analyzed due to their relevance to this study and based on the lack of use of C in the future fusion reactors. The images of all samples present an increased morphology with a textured sample surface, and the layers have a compact structure. The surface of all samples is covered in irregular shape “ridge-like” structures, which in some cases coalesce to form large clusters with irregular shape and dimensions up to several micrometers. These clusters were formed either due to the polycrystalline nature of the materials employed for the deposition or due to substrate roughness which could provide nucleation centers. Usually the polycrystalline nature of the substrate can impact the layer morphology. From our experience, thin films grown on amorphous substrates usually tend to have a smooth surface morphology and reduced crystallinity. In contrast, the films deposited on crystalline substrates have an increased degree of crystallinity forming grains that can act as nucleation centers. On the other hand, the influence of the substrate roughness on the nucleation process is somewhat straight-forward. The peaks and valleys of the surface lead directly to surface energy variations. In this case, the nucleation sites are formed more easily in peak points compared to the valleys where nucleation sites formation is limited by the diffusion rate of the atoms in the valleys. Another factor that can account for cluster formation could be the increased deposition rate of Fe compared to that of W and Cr. SEM images for samples B and C, respectively, (Figure 2) revealed the presence, on the surface, of particles ranging in a dimension of 100–700 nm. These are accounted for as materials ejected from the deposition targets. In order to obtain more information regarding the cluster structure formations, we applied image processing software (ImageJ version 1.8.0_112, National Institute of Health, Bethesda, MD, USA). Therefore, an area integration of the largest five clusters in each image was conducted in order to obtain an average dimension on the cluster structures. The highest obtained mean values were 3.81 µm^2^ and 3.15 µm^2^ for Sample A and Sample C, respectively. In the case of the central area sample, a more refined structure was observed, characterized by a reduced surface morphology and by a lower mean cluster dimension (1.8 µm^2^). For Sample B, the calculated mean cluster dimension was ~2.6 µm^2^. These results imply that the surface morphology is strongly dependent on the sample positioning during the coating process.

The cross-section images of the investigated samples are presented in Figure 2. As was expected, the coating thicknesses varied with the position of the substrates during deposition. The lowest thickness of 1650 nm was measured for Sample A, which was placed in the proximity of the tungsten anode during the coating process, and on the other hand, the highest thickness, 3950 nm, corresponded to sample B positioned near the Fe anode, which received the highest atom flux. Cross-section SEM images of sample B appeared to show a dense layer with a homogeneous microstructure and a high packing density. One of the main mechanisms that may be responsible for the coating densification is the intense bombardment of the growing layers with energetic ions provided by the Fe plasma evaporation source. 

**Figure 3:**
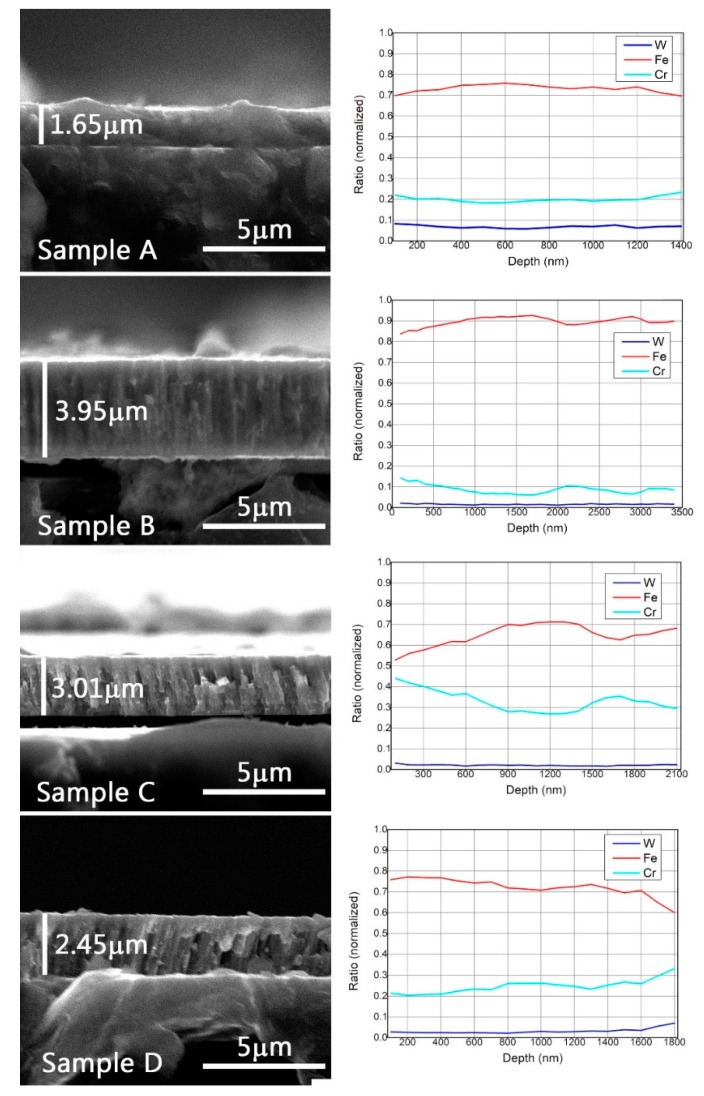
SEM cross section of samples (**left**) and EDS line analysis of cross section (**right**).

In contrast, sample C and sample D clearly exhibited columnar growth evidenced from the images in Figure 3. The Energy Dispersive X-Ray Spectroscopy (EDS) concentrations measured on the fracture zone for each sample are in good agreement with the GDOES results.

**Figure 4:**
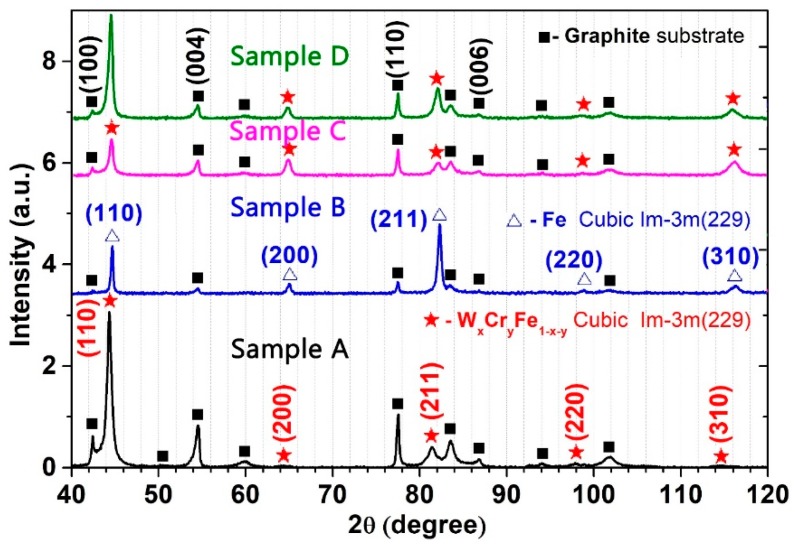
XRD patterns of W-Fe-Cr samples.

The XRD patterns obtained for the W-Fe-Cr coatings deposited on C substrate are illustrated in Figure 4. The samples deposited on C substrates were selected for this study in order to avoid the influence of the W substrate on the co-deposited W layer results. An offset has been deliberately added in Figure 4 graph for a better visualization of each sample diffraction pattern.

**Figure 5:**
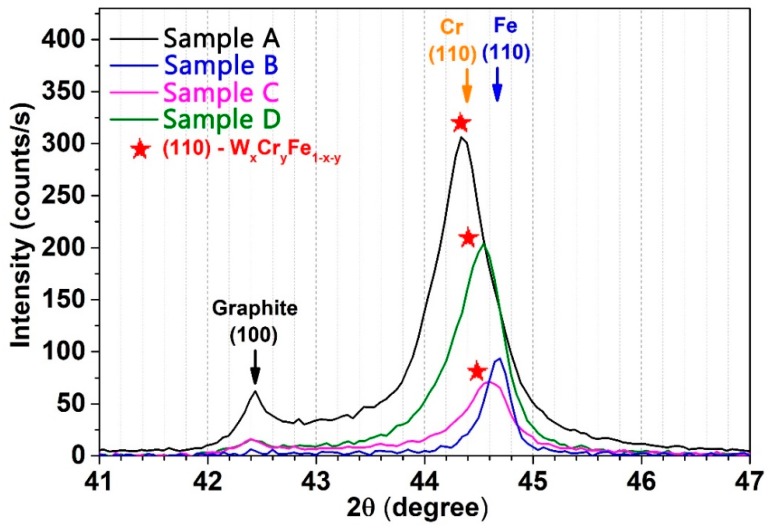
Element variation in relation to the substrate positioning during the deposition process.

The identification of crystalline phases as well as data processing was performed with specialized software named MATCH (version 3.8.3, Crystal Impact, Bonn, North-Rhyne Westphalia, Germany). The X-rays are diffracted at angles of: 42.38°, 44.52°, 54.38°, 59.78°, 64.88°, 77.53°, 83.56°, 86.75°, 101.48°, and 115.58° corresponding to various diffraction planes specific to a W_x_Cr_y_Fe_1−x−y_ alloy phase with space-group Im-3m (229) and an arrangement of atoms in a body-centered cubic lattice. The exact stoichiometry of the samples is unknown due to the lack of similar structures analyzed in literature. It is expected that the contribution of each element in the final structures varies significantly due to the substrate positioning during the coating process. This can be evidenced in Figure 5 by the different shifts of the (110)-diffraction plane. In the case of Sample B, the (110) plane was centered at a 2θ value of 44.71°, which after a careful analysis is considered to be specific to a Fe body-centered cubic crystalline phase, Im-3m (229) space group (PDF 00-006-0696). The X-ray diagram of this sample also had the highest background (due to fluorescent radiation, a phenomenon which appears when the sample contains Fe and Cu K_α_ radiation is used) which proves that it had the highest proportion of Fe. In Figure 4 and Figure 5, the background has been subtracted. This result was expected for this particular sample due to the proximity to the Fe anode during deposition and due to dominant Fe content in the coatings. Surprisingly, no Cr- or W-specific peaks were identified in the X-ray diffraction pattern of this sample. For Sample A, the 2θ position of the (110) peak has a massive shift of 0.32° to lower angles as compared to the (110) peak (44.43°) of a 100% cubic Cr sample (Im-3m (229) space group, PDF 00-006-0694), and it can be considered that this behavior was caused by the increased W content in the sample. The same behavior for the (110) peaks was observed for Sample C and Sample D, respectively, but with a lower shift (their positions are in between the positions of (110) peaks for 100% Cr and 100% Fe samples), which suggests some degree of element mixing in the coatings. By comparing the relative intensity of the diffraction peaks, it was observed that Sample A and Sample D exhibit the highest degree of crystallinity compared to the rest of the samples. Sample B also has preferential crystalline growth on (211) orientations.

The intensity of peaks corresponding to the C substrate (Figure 4) varied from one sample to another for two reasons: one may be the thickness of the samples, and the other is the fluorescent radiation. It was noticed that the XRD pattern of Sample A presents the highest intensity for peaks corresponding to the substrate. In contrast, the lowest intensities were observed for Sample B. This variation of thickness for samples deposited in fixed geometry is to be expected due to the different particle flux received from each material during the deposition, which was confirmed in this paper by means of GDOES and micro-XRF results.

The lattice constants were determined from the diffraction patterns with the help of the MATCH software. The highest value obtained was for the Sample A, where the lattice constant was 2.898 Å, which corresponds to the lowest calculated density of all samples with the value of 7.621 g/cm^3^. The values of the lattice constants for Samples C and D were 2.873 Å and 2.872 Å with the calculated densities of 7.821 g/cm^3^ and 7.834 g/cm^3^, respectively. As for Sample B, the lattice constant has a calculated value of 2.866 Å and a density of 7.873 g/cm^3^. The grain size dimensions were calculated based on the major (110) peaks for each sample using the Debye–Scherrer relation. The values for Samples A, C, and D are similar with ~10 nm grain size dimensions. The grain size dimension for Sample B was increased to 22 nm by a factor of two.

### 3.2. Compositional Analysis

In the past, the micro-XRF method was proven to determine thickness layers in the range of tens of nanometers up to 6 micrometers for the highly attenuated L energy lines of W. For the other studied elements, such as Cr and Fe, one expects a signal saturation threshold much higher than the overall thickness of the deposited layers involved in the current experiment, due to the presence of highly energetic K lines that presented a lower self-attenuation degree in the sample volume. Therefore, relevant arbitrary unit measurements could be evaluated. This method was applied on the C substrate co-depositions in order to avoid the amplification of the W layer peak intensities from the W substrate.

**Figure 6:**
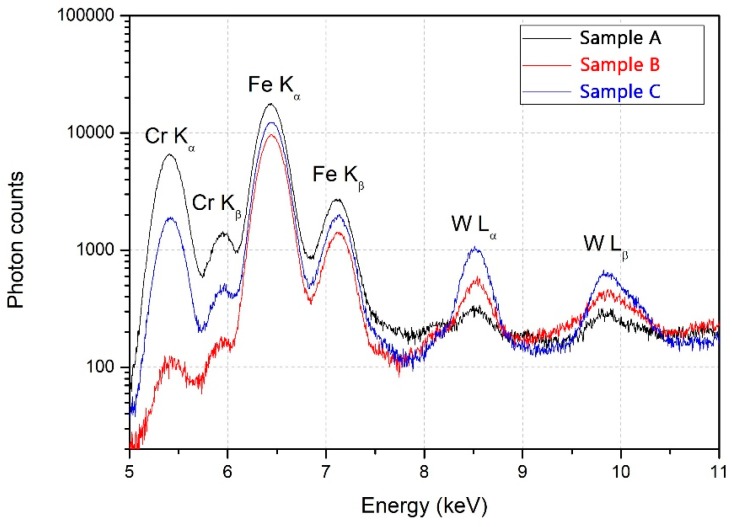
X-ray fluorescence (XRF) spectrum highlighting characteristic energetic lines for the relevant investigated elements.

In the fluorescence spectrum (Figure 6), the relevant K and L characteristic energetic lines for the investigated samples (Cr K_α_-5.41 and Cr K_β_-5.95, Fe K_α_-6.4 and Fe K_β_-7.06, and W L_α_-8.40 and W L_β_-9.67) could be measured. It is important to mention the fact that the characteristic energies for the investigated elements are slightly overlapped, therefore it is important to consider the possible analysis errors due to matrix coefficients, translated into amplified or attenuated fluorescence peak intensities.

**Figure 7:**
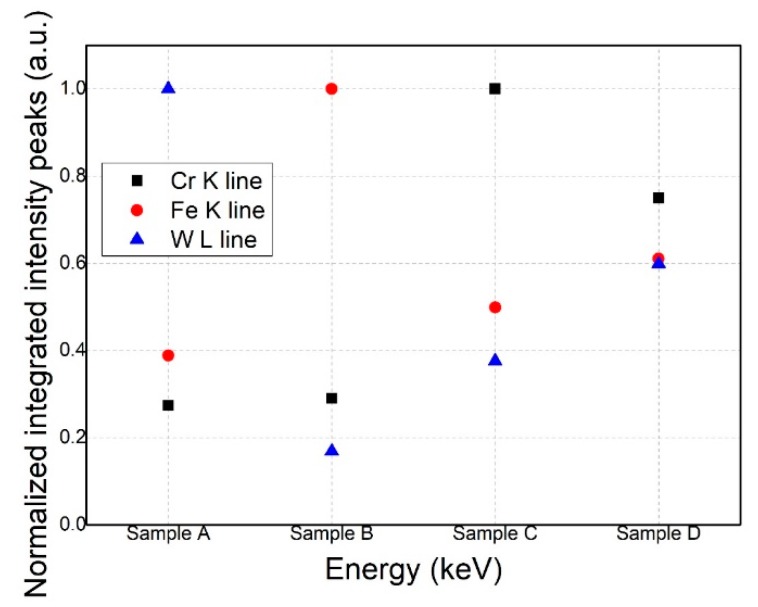
XRF results on deposited materials in relation to the spatial sample substrate distribution in the chamber.

A measurement campaign was conducted on C substrate co-deposited samples, thus resulting in the intensity variations for each element expressed in arbitrary units (Figure 7). The integrated intensity values for each element were normalized to the highest determined intensity peak.

The micro-XRF empirical results on the co-deposited alloys provided predictable data; thus, each sample that was in the direct projection of a certain anode determined a highest fluorescence signal for that specific anode material in comparison with other positions. These results come as a confirmation for the spatial distribution of the TVA, relying on the fact that in the central area of the sample holder, mean values for the X-ray fluorescence intensities for each deposited element were obtained.

For the determination of quantitative results, the XRF-FP software was applied for all experimental data measured from previous conducted mappings on Sample D. The mapping was conducted on an area of 6 × 6 mm with an investigation step of 0.5 mm, totalizing a number of 169 measured data points. The XRF-FP software implies several processing steps, such as: a powerful noise reduction, peak deconvolution, removal of escape peaks, and a series of artefact mitigation algorithms. Therefore, the correctly integrated net intensities of fluorescence peaks were converted to at% from the initially by the software determined results that were expressed in wt % or mole%. It was emphasized that for a complete elemental concentration analysis, XRF-FP software was applied without calibration samples. If the results were necessary to be expressed in thickness values, then thickness-calibrated samples were necessary.

**Figure 8:**
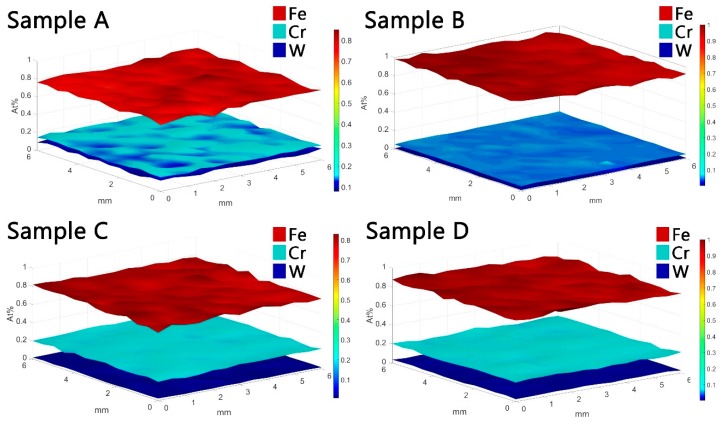
XRF-FP (fundamental parameters) based area mapping results of samples.

Mean values of at% ± errors were determined from the investigated surface for each sample: Sample A (Cr 12.7 ± 3.56, Fe 78.8 ± 8.87, W 8.4 ± 2.89), Sample B (Cr 4.1 ± 2.02, Fe 94.9 ± 9.74, W 0.9 ± 0.94), Sample C (Cr 22.3 ± 4.72, Fe 75.4 ± 8.68, W 2.1 ± 1.44), and Sample D (Cr 13.7 ± 3.7, Fe 83.6 ± 9.14, W 2.5 ± 1.58). The results obtained from sample mapping are illustrated in Figure 8.

**Figure 9:**
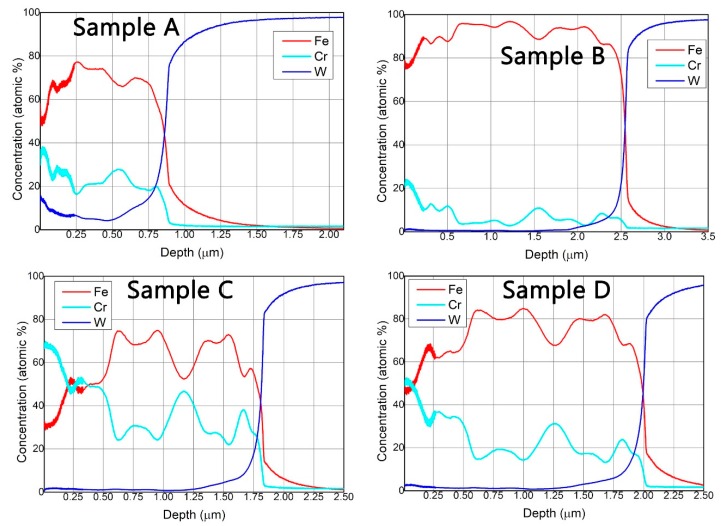
Glow discharge optical emission spectrometry (GDOES) depth profiles of samples.

In order to perform the elemental depth profile, an analysis method for GDOES had to be defined. This analysis method implied the selection of glow discharge parameters (voltage and current), calibration and recalibration samples, and other parameters involved in performing GDOES measurement. A series of certified calibration samples with known Fe, Cr, C, and W concentrations were used in the process of setting up the analysis method. In the measured GDOES spectra, differences could be observed between the co-deposition in relation to the spatial distribution of substrates on the sample holder. The typical GDOES depth profiles obtained for the analyzed W-Fe-Cr co-depositions are illustrated in Figure 9.

In the case of Sample A, as expected, the W concentration in the deposited coating was higher than in the rest of the samples with a concentration exceeding 10 at%. In this case, the Fe, Cr, and W were detected up to a depth of 900 nm. On the other hand, in the case of the Sample C, an increased Cr concentration (70 at%), even higher than Fe, was observed in the first 300 nm of the coating due to the position of the sample on the holder and due to the proximity to the Cr anode. The total thickness of the coating in this case was ~1.8 µm.

GDOES depth profiles revealed a non-uniform distribution of elements in the coatings evidenced by large variations of both Fe and Cr concentrations. This was observed for all samples due to the fact that the deposition rate varied with the plasma parameters. Keeping a stable deposition rate during the anode material evaporation process is not trivial to realize. It was observed that the evaporation rate presents a variation in the alloy depositions because it is related to factors like the cathode temperature and dissipated power on the anode, which are factors that are not straightforward to control during the deposition process. Due to the higher evaporation rate of Fe in comparison to Cr and W, a thicker deposited layer was observed for the sample that stands for Sample B, were the relevant elements were detected up to a depth of ~2.5 µm.

### 3.3. Mechanical Properties

**Figure 10:**
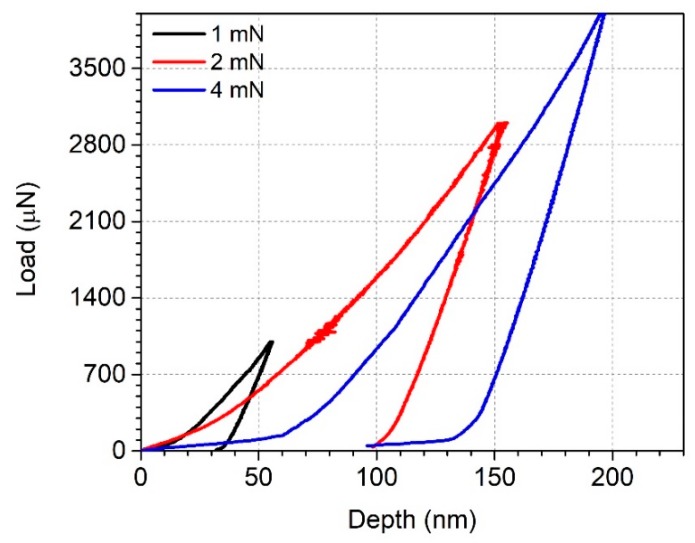
Indentation loading–unloading test at a normal load of 1 mN, 3 mN, and 4 mN.

The proposed technique to be applied in order to conduct a mechanical interpretation was the nanoindentation method. The influence of the W substrate must be minimized by adjusting the penetration depth of the nano-indenter to 10–20% of the coating thickness in order to obtain accurate information regarding the coating’s hardness and elastic modulus. For this purpose, in Figure 10, the loading–unloading test results are displayed for 1 mN, 3 mN, and 4 mN normal load obtained for Sample C. It was observed that the penetration depth increased from 50 to approximately 200 nm, for a load of 1 and 4 mN, respectively. Therefore, the selected load used for the rest of the measurements was 4 mN.

**Figure 11:**
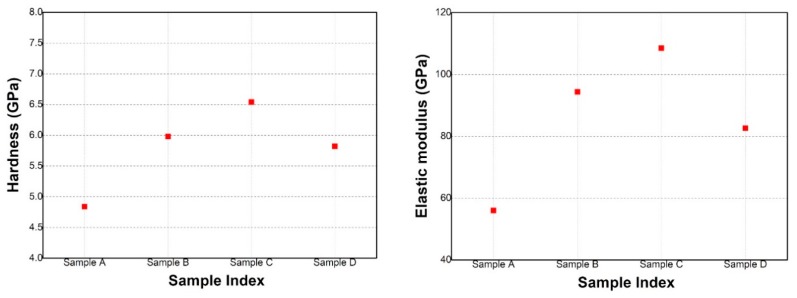
Sample hardness (**left**) and elastic modulus (**right**) obtained at 4 mN indentation load.

The average values of hardness and the elastic modulus obtained from the load–unload measurements performed at 4 mN for all W substrate deposited samples are represented in Figure 11 left and right, respectively.

The hardness and elastic modulus present a maximum value for Sample C (H = 6.84 GPa and E = 108.47 GPa) followed by Samples B and D. The lowest values for the relevant parameters were recorded for Sample A (H = 4.84 GPa and E = 56.01 GPa). Thus, ones can observe that the samples positioned above the anodes with the highest deposition rates for Fe and Cr have the highest values for hardness. A higher power applied on both anodes lead to an increased flux of ions to their corresponding samples, which led to an improved ad-atom mobility and an increase in hardness due to the densification of the coating under enhanced ionic bombardment. Another important parameter which can influence the coating hardness is the Cr concentration. As expected by these criteria, Sample C presents the highest hardness. On the other hand, the Cr concentration is higher for Sample D compared to Sample B, but the hardness is lower in value. In addition, the sample microstructure plays an important role for hardness measurements. It is very difficult to separate the contribution of each of these factors contributing to the coating hardness, and it will require further research to understand the hardening mechanisms.

**Figure 12:**
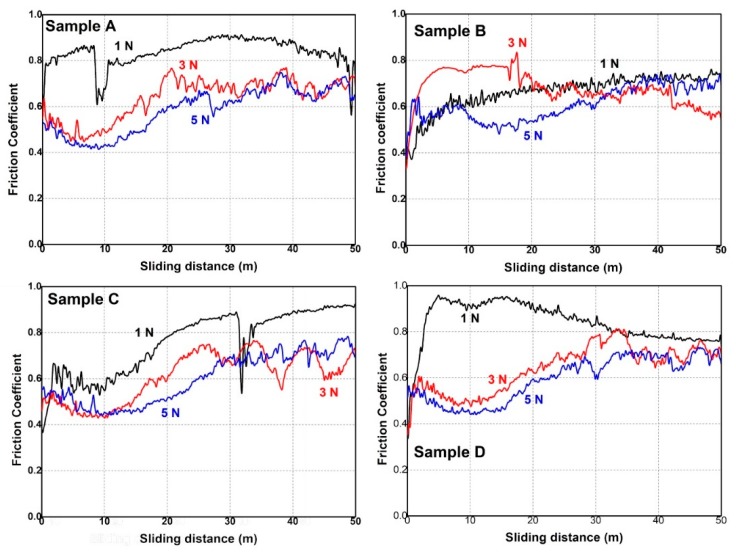
Plot of friction coefficient as a function of sliding distance in relation to normal loads of 1 N, 3 N, and 5 N.

The analysis of the sliding wear behavior of the W-Fe-Cr coatings deposited on W substrate was conducted in order to investigate the influence of the sample composition on to the friction and wear coefficients and to assess the main mechanisms responsible for sample erosion when sliding against a stationary stainless-steel ball. These measurements were performed at room temperature under normal humidity conditions. The measured specimens are illustrated in Figure 12 where the friction coefficient is represented as a function of sliding distance under 1 N, 3 N, and 5 N normal loads. 

Results obtained for 1 N load measurements for Sample B indicate a two-stage plot: a running-in characteristic described by a sharp increase of the friction coefficient and a quasi-uniform threshold. In this case, the average friction coefficient has a value of 0.65. On the other hand, Samples A and C present the running-in stage but not a steady-stage. In both cases, a sharp decrease of the friction coefficient can be observed just below 10 m sliding distance for W and approximately 32 m for Sample C. This phenomenon occurs most likely when the coating is delaminated under the intense plowing effect of the stainless-steel counter-surface. It was also observed that both coatings exhibit the same behavior mentioned above at an approximate value of 0.85 for the friction coefficient. This stage is followed by a steady increase in friction with a high contribution from the wear debris. The failure of these coatings may be caused by the reduced thickness, especially in the case of Sample A. Furthermore, delamination can occur due to the merging asperities found on the substrate. In this case, the hardness of W-Fe-Cr is subjected to a high shearing stress generated by the sliding against a hard counter-surface and the substrate asperities, which can lead to grinding of the coating. 

For Samples A, C, and D (Figure 12), a reduction of the friction was observed for the 3 N and 5 N loads, respectively, in comparison to the 1 N load. This was caused by the improved contact of the counter-surface with the coating. In this case, in the running-in stage, a gradual decrease of the friction coefficient was observed in the first 10 m sliding distance. This was followed by a gradual increase of the friction coefficient, followed by large variations of the same parameter mostly in the last 20 m of the sliding cycle. It is difficult in this case, to evaluate at which point the coating was delaminated, but the large variations of friction are caused by wear debris.

In contrast, in Figure 12 for Sample B, a very different sliding wear behavior was observed for the 3 N and 5 N loads in comparison to the rest of the samples. In the running-in stage of the measurement, the friction coefficient increased rapidly, especially for the 3 N load. In addition, the friction coefficient is higher compared to the other three samples. This behavior can be explained due to the different nature of the contact with the stainless-steel counter-surface. Therefore, the nature of the contact is abrasive due to the high Cr concentration revealed both by micro-XRF and GDOES measurements. The Cr content increases both the hardness of the coating and enhances its anti-deformation properties. In the case of Sample B, due to the high Fe concentration, the contact between the stainless-steel ball and the coating may have an adherent component due to the softness of Fe.

**Figure 13:**
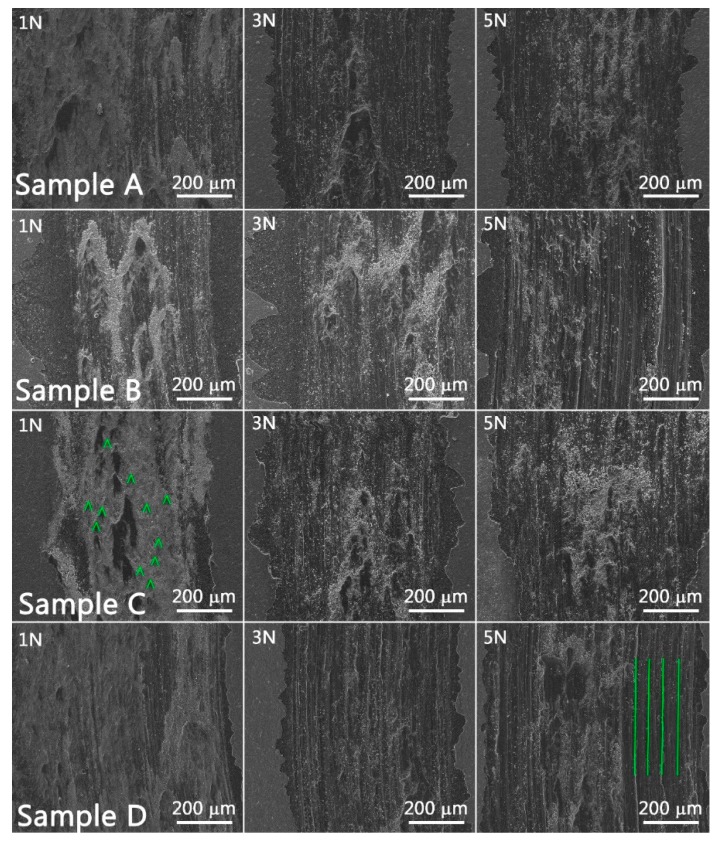
SEM images of the wear tracks formed at normal loads of 1 N, 3 N, and 5 N. Parallel grooves observed for the wear track of sample D at 5 N normal load indicated an abrasive contact between the stainless-steel ball and the coating countersurface. The adhesion zones are highlighted for the iron-rich sample at 1 N normal load.

For a better understanding of the sliding-wear behavior, SEM images were taken of the wear tracks. These images are illustrated in Figure 13 including the legend regarding the applied normal load during the measurements. The intense plowing action of the stainless-steel ball can be observed even for 1 N normal load. For example, extensive plastic deformation was observed especially for Samples A, B, and D, where the abrasive contact is revealed by the parallel ploughing grooves and ridges that were formed along the wear tracks. This was especially visible for 3 N and 5 N of applied load during measurements. On the other hand, Sample B measured at 1 N load exhibits adhesion zones and shallow grooves along the wear tracks due to the high concentration of soft material (Fe).

## 4. Conclusions

Next-generation fusion devices like DEMO and ITER rely on the development of the PFC and test blanket module. Hence, in this paper, the W-Fe-Cr based alloys were studied from a compositional, mechanical, and structural point of view. The W-Fe-Cr coatings with variable material concentrations were successfully deposited by means of the TVA method, thus validating the method as being proper for co-deposition layer fabrication. The SEM measurements revealed a high textured morphology of the surface which is covered with irregular size ‘ridge-like’ structures. From the XRD measurements, the formation of polycrystalline W_x_Cr_y_Fe_1−x−y_ alloys with a body-centered cubic lattice was revealed for all samples with the exception of Sample B which exhibited a 100% Fe body-centered cubic polycrystalline phase. The highest crystallinity was presented by Sample A. By extracting the grain size dimension from the diffraction data, an approximate value of 10 nm was obtained for all samples, with the exception of Sample B, which had a grain size of 22 nm. The compositional analysis protocol performed by means of micro-XRF method assisted with the XRF-FP software provided information regarding the elemental concentration for each deposited sample. Predictable at% data were obtained providing a quasi-uniform deposition trend on the coating surface area.

The GDOES depth profiles indicate a variation of thickness and composition in respect to sample position during the coating process. The nanoindentation measurements performed to a depth of 200 nm revealed that Sample C exhibited the highest hardness (6.84 GPa), while Sample A had the lowest hardness (4.84 GPa) of the all measured samples. The tribological measurements indicated a reduced friction for samples measured at 3 N and 5 N loads in comparison to 1 N load applied for all samples, with the exception of Sample B. In addition, the SEM images of the wear tracks revealed extensive plastic deformation and an abrasive wear behavior signaled by the parallel grooves and ridges formed along the wear track. 

## Figures and Tables

**Table 1:** Discharge parameters for the W, Fe, and Cr anode.

Deposited Material	U_a_ (kV)	I_a_ (A)	I_f_ (A)	Mean Deposition Rate (nm/s)
W	2.082	1.335	50.05	0.02
Cr	1.709	0.324	31.02	0.04
Fe	0.827	1.013	48.63	0.36

**Table 2:** Sample nomenclature according to position during deposition.

Sample Index	Sample in Proximity to Anode
Sample A	Tungsten
Sample B	Iron
Sample C	Chromium
Sample D	Central position
